# Mechanically Tunable Biofabricated Channels Enable Mimicking Arterial Pulsatility and Dynamic Tissue Actuation

**DOI:** 10.1002/smsc.202500176

**Published:** 2025-06-30

**Authors:** Cécile Bosmans, Malin Becker, Liliana S. Moreira Teixeira, Jeroen Leijten

**Affiliations:** ^1^ Leijten Lab Department of BioEngineering TechMed Centre Faculty of Science and Technology University of Twente Enschede 7522 NB Netherlands; ^2^ Department of Advanced Organ bioengineering and Therapeutics, TechMed Centre, Faculty of Science and Technology University of Twente Enschede 7522 NB Netherlands

**Keywords:** biofabrications, biofunctionalization, embedded bioprinting, tissue engineering, vascularization

## Abstract

Dynamic alteration of blood vessel geometry is an inherent feature of the circulatory system. However, while the engineering of multiscale, branched, and interconnected blood vessels has been well explored, mimicking the dynamic behavior (e.g., pulsatile blood flow) of native arterial vessels has remained understudied. This is surprising because the natural pulsatile flow and subsequent dynamic deformation of arteries provide physiologically relevant mechanical actuation to proximal cells and tissues, contributing to both tissue homeostasis and disease progression. Yet, many tissue engineering efforts and Organ‐on‐Chip developments have focused on replicating vessel structure, while overlooking the native mechanical dynamicity that governs arterial tissue function. Here, the development of an on‐demand tunable elastic hydrogel is reported, composed of tyramine‐conjugated alginate, offering controlled, reversible dilation under physiologically relevant flow. Exploring casted and 3D bioprinted channels, how vessel dilation influences shear stresses in relation to vessel compliance is investigated. This approach is demonstrated to allow for hydrodynamic mechanodeformation and stimulation of engineered tissues. Moreover, it is revealed that pulsatile flow deformation alters compound penetration rates (e.g., nutrients and pharmaceuticals) into surrounding tissues. Finally, the spatially controlled stiffening of engineered blood vessels is demonstrated to locally limit the dilation, modeling blood vessel diseases such as stenosis or aneurysm.

## Graphical abstract

A tunable hydrogel is developed to mimic arterial expansion and contraction under pulsatile blood flow, which is an often overlooked natural phenomenon. The approach enables flow‐responsive wall deformation, perivascular tissue actuation, and enhanced molecule penetration. Additionally, local mechanical disturbances are introduced to mimic vasculopathies such as aneurysms. This platform advances the capacity to model arterially perfused living matter.

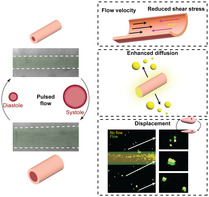

## 1 Introduction

Rhythmic cardiac pumping drives blood flow through the vascular network within tissues and organs to exchange nutrients, waste products, and endocrine compounds. This blood propagation is associated with a pulsatile pressure wave (e.g., systolic and diastolic blood pressure) from the heart to the rest of the arterial tree, which includes the arteries and arterioles.^[^ ^1^, ^2^ ^]^ The pulsatile wave propagates via elastic deformation of the arterial tissue, storing stroke volume, which is discharged during the diastole, maintaining a continuous blood flow. This elastic mechanical deformation effectively acts to activate adjoining tissues, affecting mechanosensitive cells via different mechanical cues. Smooth muscle cells in the tunica media of arterial vessels experience stretch^[^ ^3^, ^4^, ^5^ ^]^ while endothelial cells and vascular smooth muscle cells are exposed to various flow‐derived stresses including wall shear stress, channel pressure (e.g., mechanical wall stress), and tensile stress from distention. These stresses can alter cell alignment, elongation, and protein and gene expression.^[^ ^4^, ^5^, ^6^, ^7^, ^8^, ^9^ ^]^ Additionally, arterial pulsatility also modulates intracellular processes and molecular secretions of, among others, cytokines, growth factors, and matrix metalloproteinases.^[^ ^6^ ^]^ Not surprisingly, hemodynamic changes are correlated with the development of various arterial diseases, which can show effects directly in the vessel, such as in atherosclerosis, or in the adjoining tissue.^[^ ^10^, ^11^ ^]^ Altered mechanical deformation in conditions such as cervical artery dissection or cardiovascular disease can trigger perivascular adipose tissue accumulation.^[^ ^12^, ^13^ ^]^ Yet, despite its well‐known physiological importance, arterial pulsatility has been largely overlooked in in vitro models that aim to study the onset, development, or treatment of arterial diseases.

In past decades, a large number of vascularization models have been reported with artery to arteriole dimensions, yet lacking pulsatile hemodynamics. Recent advances involve sophisticated biofabrication methodologies such as 3D bioprinting of blood vessels, enhancing resolution, and complexity.^[^ ^14^ ^]^ Notable approaches include layer by layer direct bioprinting (e.g., extrusion,^[^ ^15^ ^]^ coaxial extrusion,^[^ ^16^ ^]^ digital light processing)^[^ ^17^ ^]^ or embedded bioprinting of sacrificial inks enabling freeform biofabrication of hollow structures (via extrusion^[^ ^18^, ^19^, ^20^, ^21^ ^]^ and coaxial extrusion).^[^ ^22^ ^]^ These platforms, which are proposed or used for disease modeling and drug screening, focus almost exclusively on the mechanical stiffness and shear stress of engineered arterial models.^[^ ^8^ ^]^ Moreover, studies incorporating pulsatile flow often exhibit minimal deformation and thus are still subjected to venous flow profiles from a perivascular perspective.^[^ ^15^, ^17^, ^19^, ^22^, ^23^ ^]^ To date, only a few studies have incorporated dynamic, compressive, and tensile stresses,^[^ ^14^, ^24^, ^25^, ^26^ ^]^ but these approaches still lack the mechanical tunability, spatial patterning, or fabrication robustness required for in‐depth investigations. In summary, studies focused on engineering mechanical tissue properties to enable programmable and physiologically relevant deformation for arterial pulsatility remain largely unexplored.^[^ ^27^ ^]^

Here, we developed 3D tissues with perfusable channels at the arterial and arteriole scale, with the unique ability to rapidly and reversibly deform upon exposure to flow pressure. This was achieved by functionalizing an alginate backbone with tyramine moieties (ATA), which created a system with highly tunable mechanical properties upon covalent crosslinking. ATA hydrogels could be used for the casting of perfusable channels, and for creating branching channels with aqueous two‐phase low viscous embedded 3D bioprinting, as shown in previous work.^[^ ^28^ ^]^ While the low viscous nature of the precursor material allowed for facile handling, the mechanical tunability enabled control over channel expansion under flow, which in turn offered accurate control over wall shear stresses and mechanical deformation of the surrounding hydrogel that mimics peri‐arterial tissue. Pulsatile perfusion enhanced the penetration of molecules from the channel into the tissue and accelerated their diffusion. We then leveraged ATA's mechanical tailorability to model vasculopathies associated with localized mechanical differences, such as arterial stiffening, atherosclerosis, or aneurysms, by locally altering the hydrogel's mechanical properties. This feat is anticipated to aid in investigating arterial mechanical homeostasis disruption, associated with altered blood pressure and cardiovascular complications.^[^ ^6^, ^7^, ^8^, ^29^ ^]^ Moreover, as this modular system offers ease of use and tunability to model key features of hemodynamics and vasculopathies, it can be readily integrated into existing or future microphysiological systems. This has potential biomedical applications ranging from studies of specific arterial diseases to the broader actuation of in vitro models.

## 2 Results and Discussion

**Figure 1:**
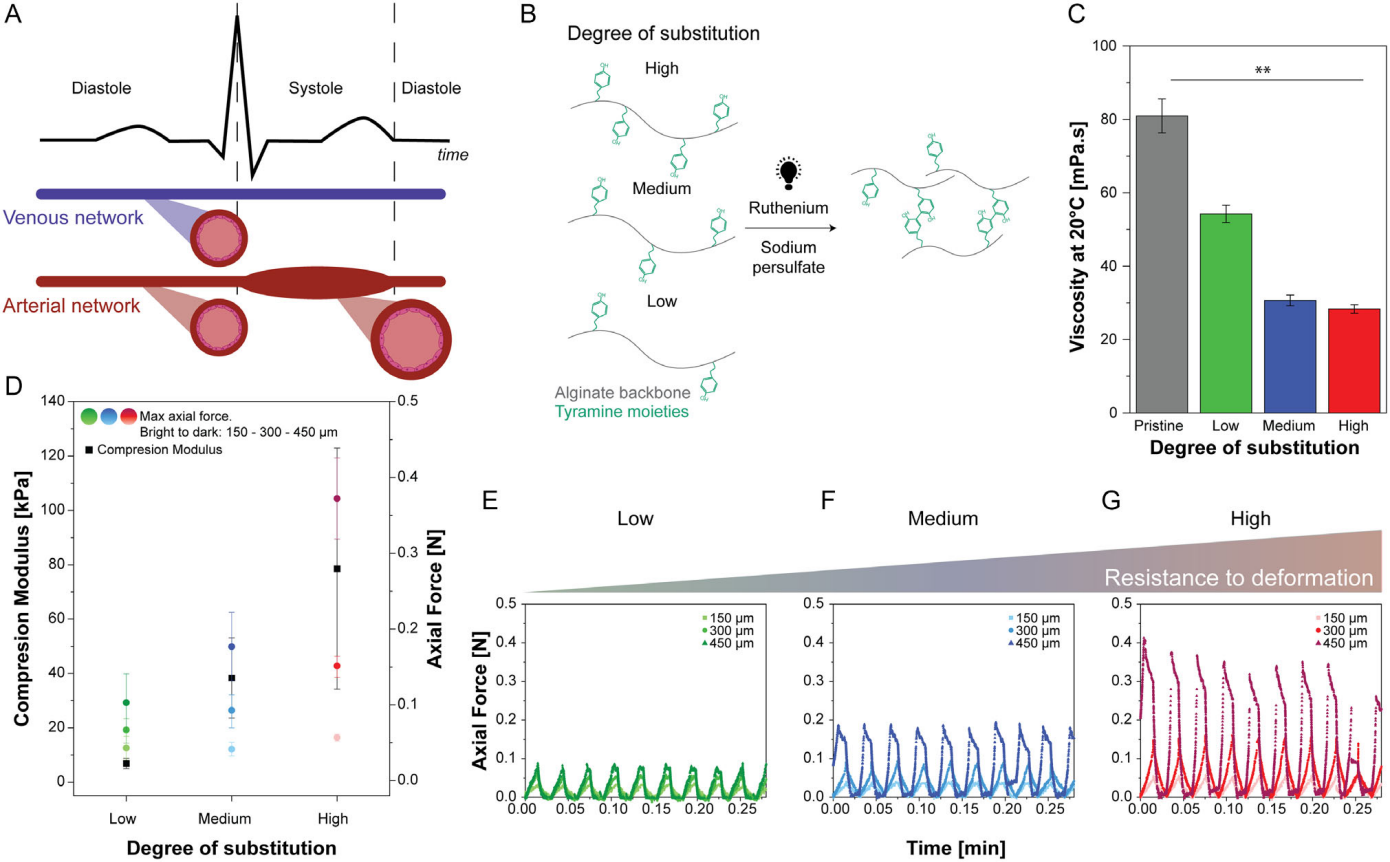
Mechanically tunable alginate–tyramine can recover from extensive elastic deformation. A) Schematic depiction of arterial and venal dynamicity, where the latter is mostly modeled in in vitro models such as organ‐on‐chip. B) Schematic depiction of tyramine functionalized alginate backbones with various degrees of substitution, as well as crosslinked alginate tyramine. C) Viscosity of pristine and functionalized alginate in dependence on their degree of substitution (Mean ± SD, *n* = 3 for each condition). D) Effect of the substitution degree on the compression modulus of alginate–tyramine gels (left axis) (Mean ± SD, *n* = 3 for each condition) and average maximal axial force required for cyclic compression of the gel at 150, 300, and 450 μm (right axis) (Mean ± SD, *n* = 4 for Low, *n* = 3 for Medium and High). E–G) Time‐resolved axial force during the cyclic compression of the alginate–tyramine gels at 150, 300, and 450 μm for low (*n* = 4) (E), medium (*n* = 3) (F) and high (*n* = 3) (G) degree of substitution materials. Statistical differences are depicted with ** significant difference p < 0.001 (1‐Way Anova Tuckey).

In contrast to venous networks, arterial blood vessels are characterized by the ability to elastically store and release energy, buffering cardiac pressure changes, and enabling continuous blood flow. This is achieved via elastic stretching of the arterial and arteriolar wall (**Figure** **1**A). Although blood vessel networks have been widely studied to investigate arterial function,^[^ ^30^ ^]^ the significant compliance of arteries and arterioles has largely been overlooked. Instead, tissue‐engineered vessels typically display venous‐like mechanical behavior, despite matching arteriole or venule size dimensions.^[^ ^31^, ^32^, ^33^, ^34^ ^]^ Yet, mechanical behavior is essential for vascular homeostasis, and local mechanical dysfunction is associated with numerous vasculopathies, including arterial stiffening, atherosclerosis, and aneurysms.

To more closely mimic the arterial mechanical compliance observed in vivo, a material must enable flow pressure‐dependent elastic stretching. Moreover, for widespread adoption, it should be easily synthesized and crosslinked, and thoroughly characterized for both mechanical and tissue engineering applications. Therefore, alginate was chosen as a versatile and easily functionalizable backbone, which upon crosslinking forms a sufficiently elastic network capable of elastic deformation under physiological flow velocities. To enable stable covalent crosslinking, tyramine groups were coupled to alginate's carboxyl groups. The degree of substitution was varied to generate softer (≈2.6%, low‐DS), medium (≈4.8%, medium‐DS), and more rigid (≈6.3%, high‐DS) hydrogel networks. ATA hydrogels were crosslinked via a photoinitiation reaction using ruthenium (Ru) and sodium persulfate (SPS) (Figure 1B). In this study, all available tyramine groups were targeted for crosslinking to highlight the material behavior as a function of its substitution degree.

Low viscosity of the hydrogel precursor is desired to enable straightforward and reproducible casting and 3D printing. Hence, we first evaluated whether functionalization impacted alginate's viscosity. The viscosity of 1% w/w synthesized ATA solutions was significantly increased when decreasing the substitution degree, showing the highest viscosity for pristine alginate (Figure 1C). This likely results from the replacement of highly charged carboxy groups by lower‐charged tyramine moieties. Here, ions present in phosphate buffered saline (PBS) might interact with the charges of the alginate, yielding higher viscosities in more charged backbones. Overall, all used solutions displayed low viscosities (<100 mPa.s at a shear rate of 10 s^−1^), and were readily compatible with casting and nozzle extrusion without bubble formation. Low viscosity is also anticipated to be beneficial for cell viability during pipetting and ejection, making the ATA hydrogel precursor suitable for most biofabrication approaches.

To assess the mechanical properties of crosslinked ATA hydrogels, bulk hydrogels were subjected to uniaxial compression. A clear increase in compression modulus was observed with increasing degree of substitution (Figure 1D, Figure S1, Supporting Information), in line with the increased availability of crosslinkable tyramine groups, which likely created a denser polymer network for the higher degree of substitution (DS) ATA. To investigate the hydrogel's recovery potential, cyclic compressions were applied, simulating pulsatile mechanical deformation. All hydrogels showed full recovery of their mechanical resistance after each cycle, as indicated by stable axial force profiles. Here, the axial force required for deformation increased with DS, matching the trend observed in compression modulus (Figure 1D,E,F,G).

Interestingly, all ATA hydrogels tolerated deformation up to ≈40% without integrity loss, further underlining the ATA's suitability for applications requiring extensive elastic deformation. As the force required to deform the hydrogels directly relates to their flow‐induced dilation behavior, the degree of substitution provides a tunable parameter to control channel compliance. Further tuning could be achieved via adjusting crosslinker concentrations (Figure S1 and S2, Supporting Information), allowing additional modulation of material stiffness even at high DS.

Overall, ATA hydrogels presented an easy‐to‐use, versatile, and mechanically tunable platform for creating elastically deforming tissue models.

**Figure 2:**
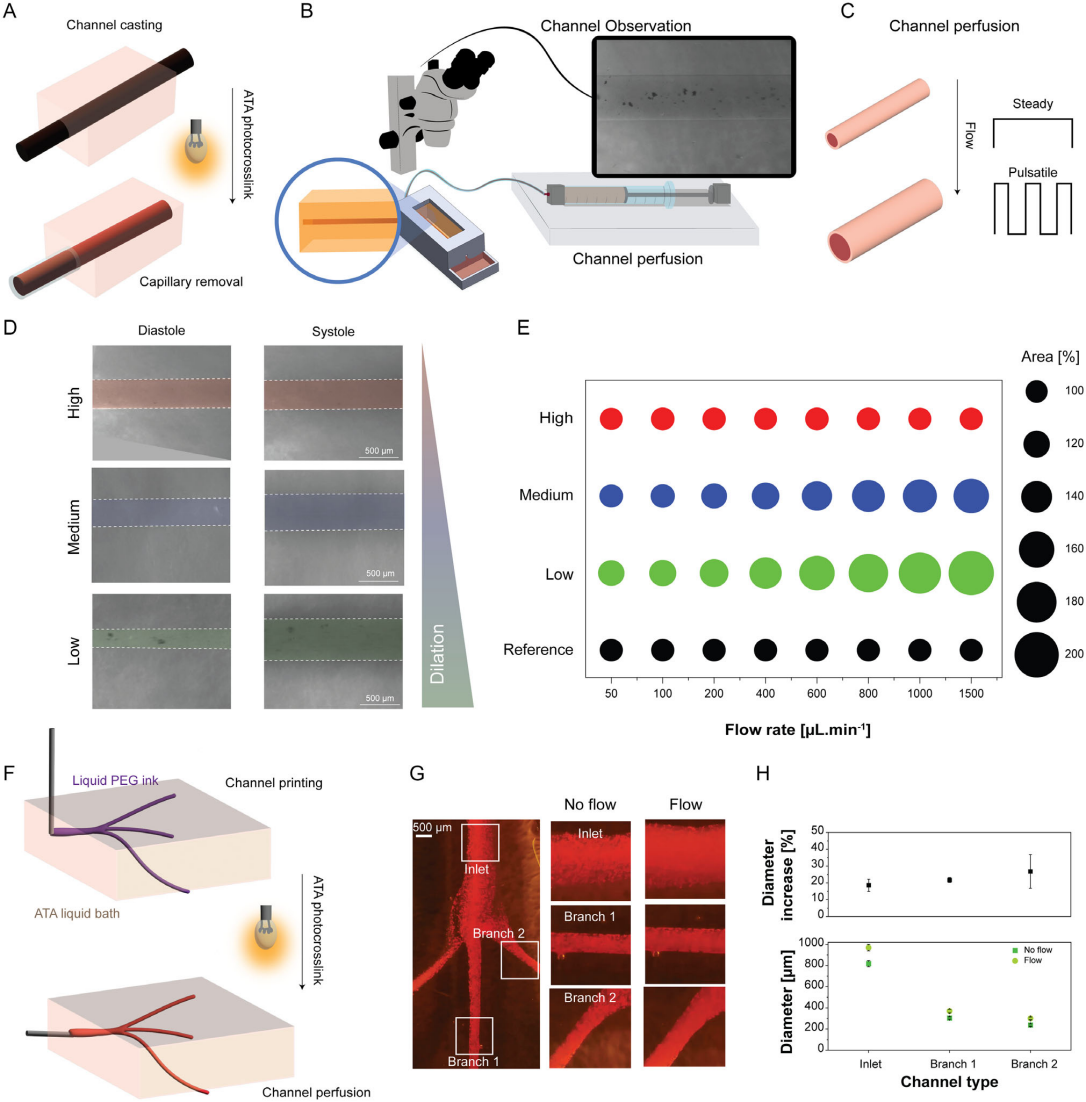
Tuning of ATA mechanical properties enables control over channel dilation upon steady perfusion. Schematic depiction of A) the template casting of the perfusable channel and B) experimental set‐up composed of the perfusable channel confined into a mold, an upright microscope for live channel observation, and a microfluidic pump for perfusion of the system. C) Schematic perfusion profiles applied in either steady flow mode or pulsatile flow mode. D) False colored images of channels under no (left) and maximal (right) flow (1500 μL.min^−1^). E) Channel area increases under steady‐perfusion of tunable‐ATA versus a reference nondilating channel, calculated from the cross‐sectional diameter of the channel (*n* = 3 channels). F) Schematic of branched channels created through low‐viscosity sacrificial printing of a PEG bioink in a low‐DS hydrogel. G) Microscope images of the created branched channels. H) Measured channel individual diameter increases upon perfusion as well as channel diameters with and without flow (Mean ± SD, *n* = 10 for each condition).

While uniaxial deformation of hydrogels provides valuable insight into material properties, the ability to deform around a channel must be thoroughly investigated to understand behavior under physiological‐like loading. Upon channel expansion, the surrounding material experiences radial compression and displacement, resulting in a force distribution distinct from uniaxial compression. To investigate this, linear channels were created using a templated casting approach where liquid ATA was crosslinked around a glass capillary to generate channels with a highly controlled initial diameter (**Figure** **2**A). The channels were confined within a mold, designed with a larger inlet and outlet, concentrating the highest pressure within the ATA surrounding the channel. The latter was perfused under distinct flow profiles and at controlled flow rates using a microfluidic pump, and real time diameter changes were monitored via upright microscopy (Figure 2B). Channel dilation upon perfusion (analogous to systole‐induced expansion, Figure 2C) revealed clear DS‐dependent differences (Figure 2D). Low‐DS hydrogels exhibited pronounced diameter increases, while medium‐DS showed moderate dilation, and high‐DS showed no apparent changes. This trend corresponds to the bulk mechanical behavior observed earlier, where higher DS resulted in stiffer hydrogels. These results indicated that control over channel dilation can be achieved by controlling both flow and material properties using fully elastic hydrogels.

To further quantify this behavior, we screened for the cross‐sectional area increase at flow rates ranging from 50  to 1500 μL.min^−1^ for all ATA backbones, using a non‐dilating (rigid) channel as a negative control (Figure 2E). Low‐DS channels showed a clear dependency on the flow rate, resulting in a near twofold increase in area for the highest flow rate (97.7% ± 4.7% area increase at 1500 μL.min^−1^). The medium‐DS hydrogel followed the same trend, however, less pronounced, with a maximal change of 26.9 ± 20.2%. The highly crosslinked network (high‐DS) created a hydrogel surrounding that was non‐compressible by the applied flows (1.3 ± 2.4% area increase at 1500 μL.min^−1^). These findings confirmed that channel dilation can be precisely controlled by the density of the created network and the applied flow rate. This tunability was further substantiated by creating partially crosslinked ATA hydrogels, where all DS were anticipated to result in looser hydrogel networks and which showed a clear flow rate/dilation correlation even in high‐DS formulations (Figure S3, Supporting Information). Additionally, reversing flow direction by applying suction from the outlet interestingly induced channel contraction (Figure S5, Supporting Information), providing an additional parameter to control channel geometry dynamically.

To extend these findings to multiple biofabrication settings and more complex architectures, low‐viscosity polyethylene glycol (PEG, 5%) was used in sacrificial printing to fabricate branched channels within low‐DS ATA hydrogels (Figure 2F). The printed structure included a main inlet bifurcating into three side branches (Figure 2G). Upon perfusion, both the inlet and side branches stably dilated, with smaller side branches showing more pronounced diameter increase (Figure 2H). This can be attributed to elevated pressure in smaller diameter channels, exerting higher forces onto the channel walls. Overall, varying channel diameters (Figure S6, Supporting Information) within hydrogels of different DS allow for precise control over channel dynamics, effectively replicating the mechanical behavior observed during diastole–systole cycles. ATA hydrogels thus offer a toolbox to tailor mechanical conditions for specific in vitro models.

While steady‐state flow may be relevant for some in vitro models, in the cardiovascular system, it is only observed within capillary and venous networks.^[^ ^9^, ^35^ ^]^ In arteries and arterioles, cardiac contraction generates a propagating wave of flow, inducing a pressure‐driven dilation wave (e.g., compliance). Ultimately, this reduces both flow velocity and pressure within the channel. To assess whether ATA channels could replicate this behavior, channels were perfused with pulsatile flow at 0.5 Hz, alternating between a low baseline (5 μL.min^−1^) and varying peak flow rates.

**Figure 3:**
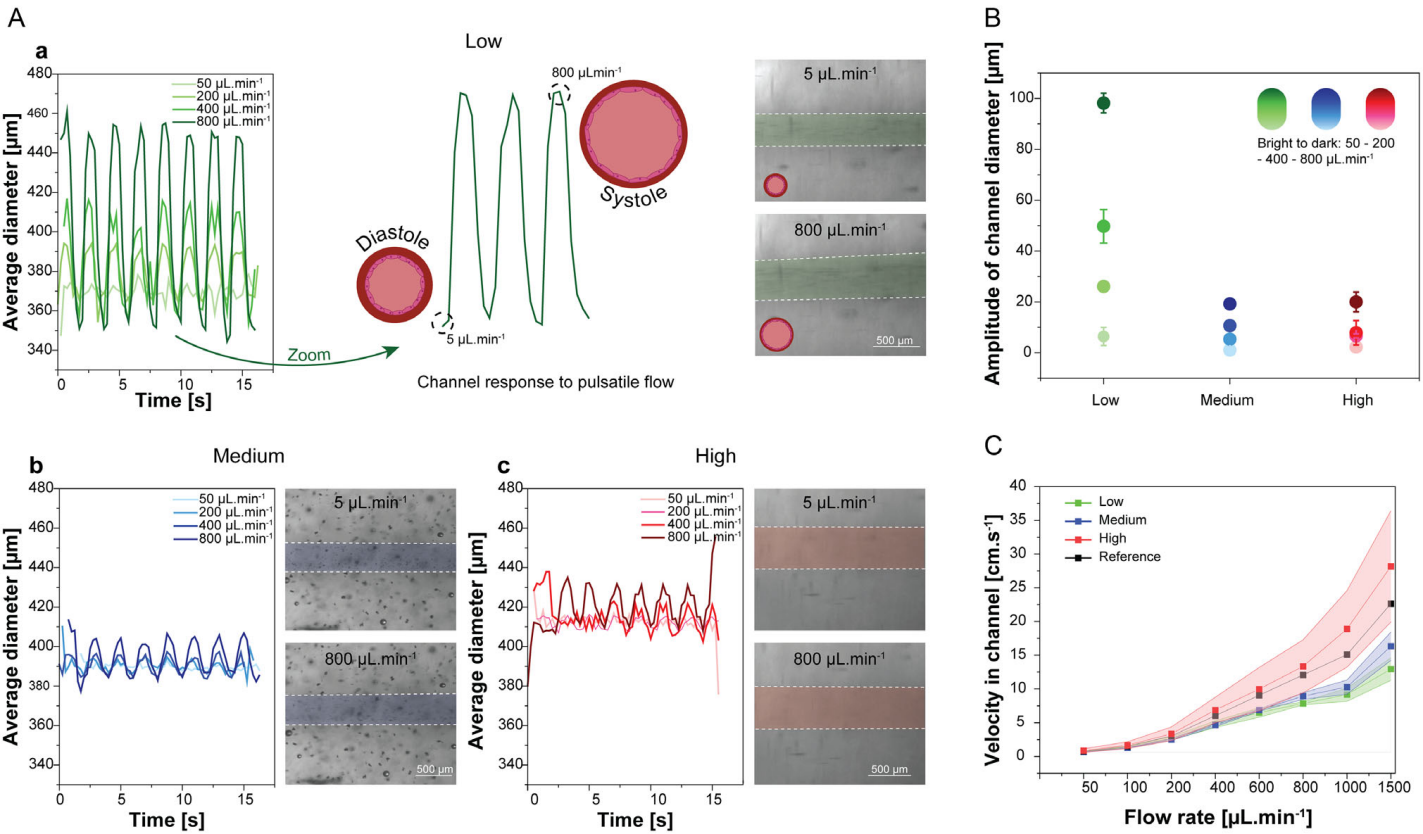
Controlled chemical functionalization allows for the creation of channels with tunable dilation properties. A) Quantification of the average diameter of a channel under pulsatile (0.5 Hz) perfusion in a) high‐dilating ATA, b) medium dilating ATA and c) high dilating ATA with increasing flow rates (from 5 μL.min^−1^ to 50, 200, 400, or 800 μL.min^−1^) with exemplary images at 5 μL.min^−1^ and 800 μL.min^−1^ (false colored) (*n* = 3 channels for each condition). B) Average maximal amplitude of the channel diameter under the different pulsating intensities for the different DS (Mean ± SD, *n* = 14 pulses for Low, *n* = 13 pulses for Medium, and *n* = 11 pulses for High). C) Calculated velocity in the channel based on calculated channel geometry at input flow rate for the tunable ATA versus a nondilating reference (Mean ± SD, *n* = 3 channels).

For all hydrogels, the amplitude of channel extension was dictated by the flow rate (**Figure** **3**A). In agreement with previous steady‐state results, lower network densities enabled greater deformation. Interestingly, across all ATA materials, channels consistently recovered to their baseline diameter between pulses when peak flows ranged from 200 to 800 μL.min^−1^. However, at a lower peak of 50 μL.min^−1^, incomplete recovery was observed: channel diameter remained larger during the low‐flow phase, possibly due to the absence of pulsatile dilation for this flow condition. As the force generated by 50 μL.min^−1^ flow was insufficient to dilate the channel wall, and no pressure release was generated upon flow rate decrease. This behavior indicates a threshold effect for pulsatile deformation. Overall, quantitative analysis confirmed that dilation amplitude under pulsatile flow scaled with both flow rate and DS, with low‐DS hydrogels exhibiting the largest deformation (Figure 3B). After pulsatile perfusion, channels fully retrieved their initial size, once again highlighting the ATA's elastic behavior and ability to rapidly recover, which is deemed crucial for cardiovascular in vitro models.

Due to elastic wall movement during pulsatile flow, the instantaneous flow velocity also varied. Using measured channel diameters at maximum extension and the applied flow rates, average internal velocities were calculated according to Equation (1) (Figure 3C, Figure S4, Supporting Information)

(1) $$v=\frac{Q}{A}$$

where *v* is the velocity in cm.s^−1^, *Q* is the flow rate in cm^3^.s^−1,^ and *A* is the cross‐sectional area of the channel in cm^2^.

Compared to non‐dilating controls, dilating ATA channels exhibited reduced internal flow velocity during peak extension, mimicking the natural vascular behavior (Figure 3A,C). This makes the ATA highly relevant for in vitro models, such as those used to regulate cell behavior at the vessel walls and in proximity to the vessels. The increased velocity in the high‐DS channels compared to the rigid control channel can potentially be attributed to a shrinking of the hydrogel, resulting in smaller diameters than the theoretical rigid control, hence, increasing the flow velocity with the same applied flow rate.

**Figure 4:**
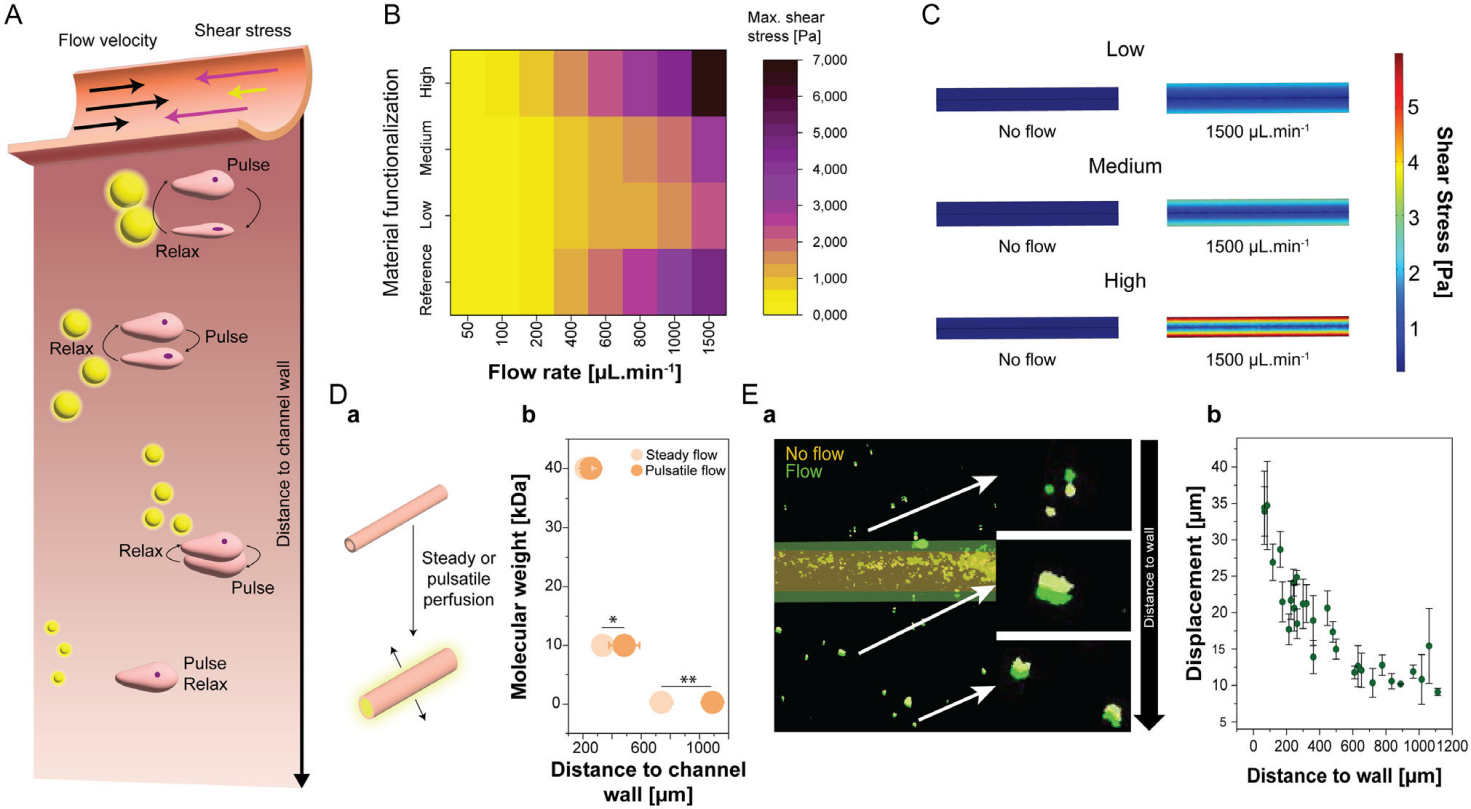
Channel dilation strongly affects stresses experienced by cells in channel proximity. A) Schematic depiction of shear forces and displacement/compression that can be experienced by cells close to the channel wall compared to the surrounding bulk. B) Maximum shear stress subjected to the wall of the differently dilating ATA channels under different rates of flow (*n* = 3 channels). C) Modeled shear stress within dilating (low) and rigid (high) channels with and without flow D) Penetration of fluorescent molecules into the bulk with a) schematic showing the effect of perfusion and b) quantifications of penetration depth of fluorophores with different molecular weights (Mean ± SD. Steady, 0.3 to 40 kDa: *n* = 8, *n* = 9, and *n* = 8. Pulsatile: *n* = 4 for each condition). E) a) Tracking of embedded particles displacement in proximity to the highly dilating channel wall and b) quantification of their displacement under pulsatile flow based on their original distance to the channel's wall (Mean ± SD, *n* = 30 positions measured at 3 distinct pulses). Statistical differences are depicted with ** significant difference p < 0.001 and * significant difference p < 0.05 (1‐Way Anova Tuckey).

While a general flow rate can be ascribed to blood flow within vessels, the velocity is not uniform but rather follows a velocity field, with lower speeds near the vessel walls leading to increased shear stress (**Figure** **4**A).^[^ ^36^, ^37^, ^38^, ^39^ ^]^ As shear stress directly correlates with flow velocity, it reaches its maximal value at higher flow rates. However, when a channel dilates under flow, the effective flow rate decreases, reducing shear stresses at the walls. These changes are known to alter various cellular behaviors essential to vessel function, including epithelial function, cell‐junction tightness, and cellular alignment.^[^ ^40^, ^41^ ^]^

To investigate how channel dilation affects maximal wall shear stress in the ATA hydrogel, a COMSOL model was employed. Simulations showcased that it was effectively reduced in highly dilating channels compared to low/no dilation (high‐DS, rigid reference), even at increasing flow rates (Figure 4B). This behavior was also supported by simulating the local shear stress distribution within the channels, confirming localized stress amplification in non‐dilating channels, whereas dilating channels exhibited a more homogeneous stress field (Figure 4C).

Beyond the channel wall, pulsatile perfusion induces mechanical deformation within the surrounding tissue via (de)compression.^[^ ^1^ ^]^ We hypothesized that such hydrodynamic actuation could enhance molecular transport. To this end, fluorescent molecules of varying molecular weights were perfused through low‐DS channels under static and pulsatile conditions (Figure 4D). The molecular weights of the fluorescent molecules were chosen to represent small molecules, such as nutrients and waste products (<1 kDa), as well as smaller (<10 kDa) and larger (<40 kDa) proteins, reflecting the size of, e.g., growth factors.^[^ ^42^ ^]^ Under pulsatile flow, all molecules exhibited deeper penetration compared to static conditions, with the effect being significant for smaller molecules but less pronounced with increasing molecular weight, showing no significant difference for molecules at 40 kDa.

The differences in penetration can potentially be attributed to transient hydrogel network expansion, increased local pressures, and convection‐like effects during relaxation phases. This enhanced diffusion profile could be highly beneficial for, e.g., nutrient and oxygen supply into the tissue, as well as facilitate indirect cell–cell communication via growth factors. This may be especially valuable in dynamic tissue engineering contexts, where efficient delivery of nutrients and oxygen is critical for cell viability and function.

Next to facilitating the penetration of molecules of varying sizes, mechanical forces are exerted on, e.g., cells in the tissue proximal to the channel wall. Particle tracking analysis revealed a near‐linear inverse correlation between displacement magnitude and distance from the channel wall, with deformation effects detectable up to ≈700 μm (Figure 4E). This mechanical gradient could be exploited to encode spatial information within in vitro models by radiating mechanical cues from pulsatile (arterial) channels, effectively mimicking the physiology of the human body. This spatially distributed actuation could also be harnessed to guide tissue organization or stimulate mechanosensitive cells in engineered constructs, broadening the platform's relevance to mechanobiology studies and biomechanically active tissues such as cardiac, musculoskeletal, or gastrointestinal models.

**Figure 5:**
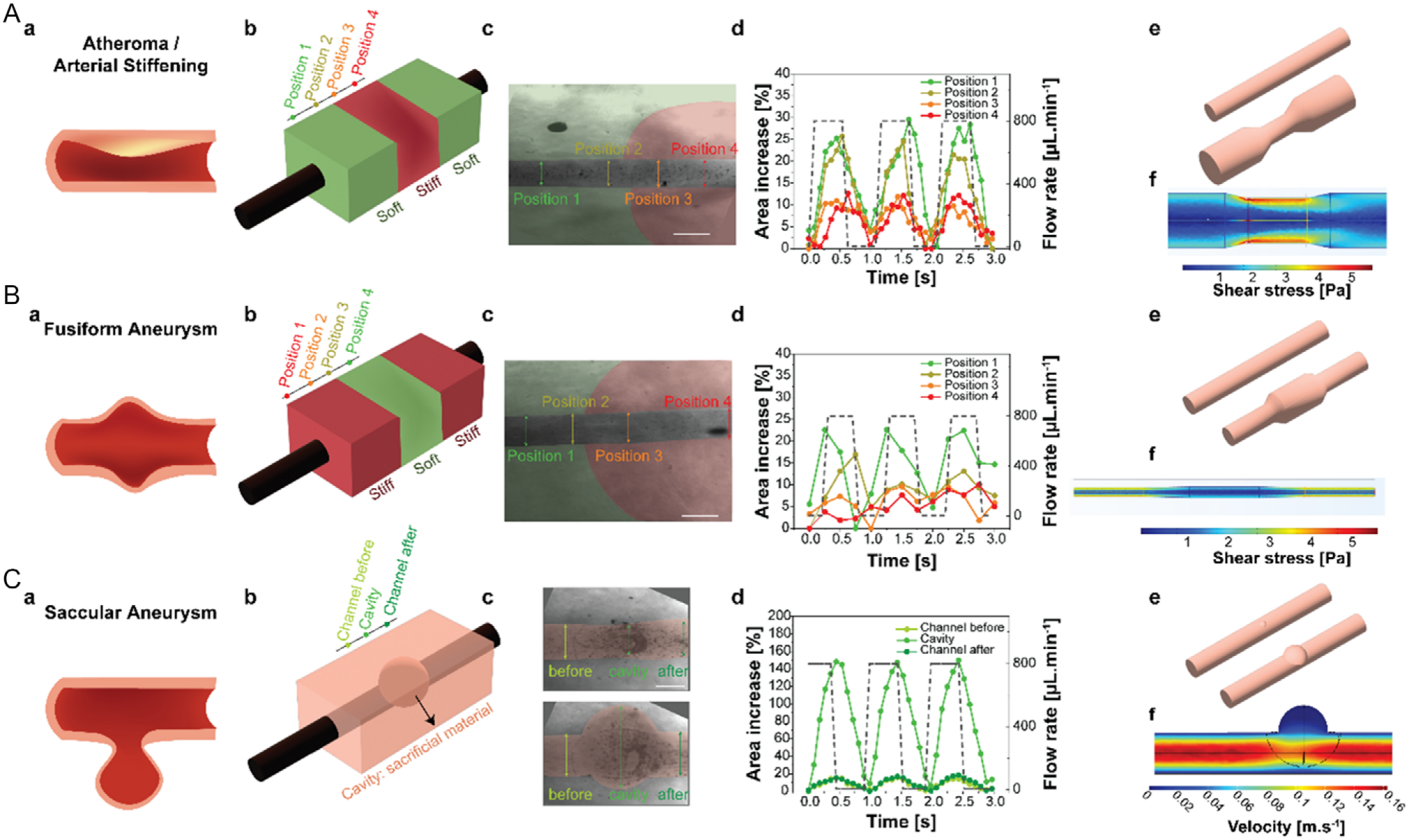
Control over (local) channel dilation can be leveraged to create models with local heterogeneities inherent to arteriopathies. A‐a) Arterial stiffening observed in atheroma or arterial stiffening and b,c) modeled through introducing a low‐dilating ATA (stiff) into a highly dilating ATA (soft). d) Dilation was observed and quantified along the heterogeneous channel (*n* = 4). e,f) The measured channel dimensions were used as an input to calculate the subjected shear stress along the stiffened arterial model. B‐a) Inversely, a fusiform aneurysm characterized by bulging of both sides of the arterial wall was b,c) modeled through the introduction of a high‐dilating ATA (soft) within a low‐dilating ATA (stiff). d) Dilation was observed and quantified along the heterogeneous channel (*n* = 4). e,f) The measured channel dimensions were used as an input to calculate the subjected shear stress in the fusiform aneurysm model. C‐a). Saccular aneurysm is characterized by bulging of one side of the arterial wall which b,c) was modeled through the introduction of a sacrificial material on top of the channel, forming a cavity linked directly to the channel. d) Dilation was observed and quantified along the heterogeneous channel (*n* = 3). e,f) The measured channel dimensions were used as an input to calculate the subjected shear stress in the saccular aneurysm model. Scale bars indicate 500 μm.

As mechanical changes in the vessels are central to many vasculopathies, such as arterial stiffening, we hypothesized that tunable material dilation could be used to model local stiffening phenomena (**Figure** **5**A‐a). To this end, channels were fabricated by sequentially casting highly dilating (low‐DS) and rigid (high‐DS) ATA hydrogels, generating modular constructs with alternating compliant and stiff segments (Figure 5A‐b,c). The channel remained straight at rest, but upon exposure to flow, reversible constrictions emerged at the stiffened area, reflecting reduced compliance (Figure 5A‐d,e). Simulations demonstrated that this constriction elevated wall shear stress locally within the stiffened zone (Figure 5A‐f).

Extending this approach, we modeled fusiform aneurysms (bulging of the two channel walls) by introducing mechanically weak regions (dilating) within a more rigid channel (Figure 5B‐a,b,c). Upon pulsatile perfusion, the channel diameter of the softer portion expanded more extensively in a flow speed‐dependent manner (Figure 5B‐d,e). Simulations confirmed corresponding localized alterations in flow velocities and shear stress distributions within the aneurysmal region (Figure 5B‐f), mimicking key mechanical features of fusiform aneurysm progression.

We further adapted the system to model saccular aneurysms, characterized by localized bulging on one side of the vessel wall (Figure 5C‐a). This was achieved by locally depositing a non‐crosslinkable liquid on top of the channel during casting, creating a structurally weak site (Figure 5C‐b,c). Upon flow‐induced pressurization, the channel formed a flow‐dependent bulbous deformation, replicating saccular aneurysm morphology (Figure 5C‐d,e). Simulations confirmed altered local flow profiles and elevated shear stresses at the site of deformation, consistent with what is reported in the literature (Figure 5C‐f).^[^ ^43^ ^]^ In addition, it is important to note that this behavior not only affects the cavity itself but also influences surrounding areas by actuating tissues in a spatially distinct manner. In the presence of cells, this could lead to changes in shear stresses experienced by the cells and contribute to clot formation when blood circulates within the cavity (Figure S7, Supporting Information).

Together, these three proof‐of‐concept models demonstrate that ATA hydrogels can be easily combined to incorporate key mechanical alterations of vascular diseases. This approach offers a novel material toolbox to replicate and study the behavior of arteriopathies as well as guide cell and tissue behavior through spatially programmable control of channel compliance, shear stress distribution, and wall deformation.

## 3 Conclusion

Alginate conjugated with tyramine was introduced as an easy‐to‐fabricate, moldable, and printable material that can be used to control mechanical compliance and facilitate channel dilation. Channel dilation was demonstrated to be inversely correlated with the amount of tyramine per backbone as well as their degree of crosslinking. For mechanically compliant materials, channel dilation was directly proportional to the applied fluid flow. Importantly, pulsatile perfusion enabled cyclical channel dilation, which was demonstrated to hydrodynamically actuate tissues and thereby facilitate penetration of molecules into the tissues. Furthermore, by combining dilating and non‐dilating material formulations, channels with locally controlled dilation or constriction can be created, which was leveraged to emulate vasculopathies, such as arterial stiffening and various aneurysms. The introduction of tyraminated alginate represents a versatile and easy‐to‐use biomaterial for generating vascular in vitro models that are force sensitive in terms of channel dilation and tissue actuation, closely mimicking the natural biomechanical behavior of in vivo arterial tissues compared to current in vitro models. Beyond vascular disease modeling, the actuation capabilities of this system may also support dynamic culture environments through physiologically relevant mechanical cues. This flexibility broadens its applicability to advanced in vitro systems such as organ‐on‐chip platforms.

## 4 Experimental Section

4.1

4.1.1

##### Synthesis

The carboxy groups of an alginic acid backbone were coupled with tyramine (Alginate‐TA) via a one‐step synthesis route. Briefly, alginic acid sodium salt (5 g, 80–120 cps) was dissolved in MilliQ water (500 mL). To achieve varying tyramine substitutions, alginate was reacted with 4‐(4,6‐dimethoxy‐1,3,5‐triazin‐2‐yl)‐4‐methyl‐morpholinium chloride at varied concentrations (DMTMM, either 12.5 or 25 mm) together with Tyramine HCl for 24 h, to yield low substitution (Low‐DS, 12.5 mM) and medium substitution (Medium‐DS, 25 mm). To achieve a high degree of substitution (High‐DS) the alginate was allowed to react for two consecutive 24 h periods in the presence of DMTMM (25 mm) and tyramine HCl (25 mm). Reactions took place at room temperature. For purification, solutions were mixed with saturated Sodium Chloride (NaCl, 40 mL) and precipitated in ice‐cold ethanol, left to sediment overnight at 4°C, and vacuum dried after filtration. The final product was obtained after dissolving in a minimal amount of MilliQ water and dialysis against MilliQ water (Spectra/Por, MWCO 1 kDa) for 4 d and subsequent lyophilization. UV–Vis spectroscopy (NanoDrop ND‐1000 spectrophotometer, 275 nm) was used to validate the correct amount of tyramine moieties per 100 repetitive units, which was ≈2.6, ≈4.6, and ≈6.3 for low, medium, and high DS ATA, respectively.

##### Crosslinking

A ruthenium (Ru) and sodium persulfate (SPS) based photoinitiator system (Advanced Biomatrix) was utilized for visible light photo‐crosslinking of the alginate backbone. To achieve different degrees of crosslinking, the material was dissolved at 1% in 1X PBS, then mixed with either high crosslinker (CR) concentration (High‐CR, 1/10 mm Ru/SPS) or low crosslinker concentration (low‐CR, 0.5/2.5 mm Ru/SPS). The material was irradiated with a Stroboscopic LED lamp (Monarch Instrument) for 3 min. Ru was typically dissolved at 0.1 m, and SPS at 1 m. For a 1 mL ATA solution, High‐CR material was prepared by adding 10  μl of SPS solution, vortexing, followed by the addition of 10 μl of Ru solution, vortexing and subsequent casting. For Low‐CR materials, preparations involved 2.5 μl SPS and 5 μl Ru solutions.

##### Material Characterization

The bulk mechanical properties of ATA with varying degrees of substitution and crosslinker concentration were assessed using rheological measurements (Anton Paar Physica MCR 301, TA Instruments HR 20). The viscosity of precursor solution was analyzed using a cup setup by screening the viscosity at shear rates from 0.01 to 100 s^−1^ and over time (120 s) at a constant shear rate of 10 s^−1^ (*N* = 3). For bulk measurements, hydrogel pellets were created by crosslinking the material in a Teflon mold. Crosslinked hydrogels were placed on the rheometer in a plate‐to‐plate geometry (8 mm) compressed cyclically (0.5 Hz) with an amplitude of 150 μm, 300 μm, and 450 μm while monitoring the normal force exerted by the hydrogels. The hydrogels were compressed at a rate of 10 μm.s^−1^, and a linear fit was applied for two strain regimes (10%–20% and 30%–40%) to assess the compression modulus of *n* = 3 samples per condition.

##### Casting Mold and Perfusion System

The channel was obtained by casting the material around a capillary of outer diameter 375 um (small channel) or a 21‐gauge needle of outer diameter 819 μm (large channel), crosslinking the material, and then removing the capillary/needle. The material was contained in a 3D‐printed rectangular mold with inlet and outlet holes (Form 3B+ stereolithography printer, Formlabs with Clear V4 resin, Formlabs). The system was perfused with a flow‐controlled syringe pump (Cetoni GmbH), glass syringe, Luer‐locks and connectors (Hamilton), tubing, and a 21‐gauge needle at the inlet. Scripts for the automatic perfusion were written in the pump's software (Cetoni ELEMENTS) to instruct volumes and duration of flow.

##### 3D Printing of Channels

For the printing of branching channels in the ATA bulk, aqueous two‐phase low‐viscous printing was applied.^[^ ^28^ ^]^ In short, PEG (5%, 35 kDa) was utilized as ink and was deposited through a 25‐gauge nozzle utilizing a microfluidic pump (Harvard Apparatus, PHD Ultra) into an ATA bath (1%) containing Ru/SPS crosslinker (1/5 mM). The desired shape was achieved by connecting the nozzle to an Inkredible and printer (CELLINK) and applying a handwritten G‐code. Directly after printing, the bath was solidified by irradiation with visible light. For channel visualization, a perfusate of 1X PBS solution with food dye and particles was used.

##### Imaging Set‐up

Imaging was performed with a stereomicroscope (Nikon), camera (IDS), and stroboscopic LED lamp (Monarch Instrument). Each system was captured with the capillary/needle for dimension reference before removal and perfusion.

Steady perfusion (Low and High‐CR; Low, Medium, High‐DS): The system was recorded and sequentially perfused at a set steady flow rate for 30 s, then 15 s recovery at 50, 100, 200, 400, 600, 800, 1000, 1500, and 2000 μL.min^−1^. The diameter of the channel was consequently measured based on the average of 10 measurement points per flow rate and per biological replicate (*n* = 3 channels), averaged per condition. From this, the area increase of the channel when it was filled in comparison to the non‐perfused channel dimensions was calculated. From the flow rate and channel cross section dimension, the velocity within the channel and, therefore also model shear stress values were obtained.

##### Pulsatile Perfusion (High‐CR; Low, Medium, High‐DS)

The system was recorded and sequentially perfused at 50 μL.min^−1^ for 1 min, 20 times at a given flow rate for 1 s, then at a 5 μL.min^−1^ for another second for the following flow rate values: 50, 200, 400, 800 μL.min^−1^. For each given flow rate, the diameter of the channel was consequently measured every 25 microseconds based on the average of 10 measurement points, per created channel, averaged per condition. From this, the channel diameter over time per flow rate and per condition was plotted. The pulse amplitude of the channel dilation was then calculated based on the average of pulses per flow rate and per condition (*n* = 3 channels). Particles in the channel surrounding were tracked for pulsatile perfusion (5 and 800 μL.min^−1^) using ImageJ. For particle displacement around the channel wall, particles were directly mixed in the material before crosslinking. Particles at varying distances were then tracked for Low‐DS, High‐CR material, and their distance to the channel wall was measured over 3 pulses and averaged.

##### Diffusion (High‐CR; Low‐DS)

The system was recorded and perfused under steady or pulsatile mode at equivalent volumes (for 10 min at 202 and 400‐5 μL.min^−1^, period = 2 s) using fluorescent solutions of different molecular weights: 0.379 (fluorescein sodium salt, Sigma), 10 and 40 kDa (fluorescein isothiocyanate‐dextran, Sigma). The intensity was then measured around the channel wall, and the diffusion distance was measured at different locations along the channel and averaged for each condition.

##### Engineering of in vitro *vasculopathy Models*

Low and high‐DS materials were used together to cast around the capillary to obtain heterogeneous channel dilation under flow. For arterial stiffening (atheroma/arterial stiffening modeling) and softening (fusiform aneurysm), Low‐DS material was cast around the capillary, and a drop of High‐DS material was placed on the capillary (or reversed). For saccular aneurysm modeling, Low‐DS material was cast around the capillary, and a drop of material without crosslinkers was placed on the capillary, and therefore was washed away when the channel was perfused. After crosslinking, we recorded the interface of the materials when perfused in pulsatile mode (50, 400, and 800 μL.min^−1^) and measured the diameter of the channel at distinct positions. The average area increase per position was calculated, which allowed for estimation of shear stress or velocity magnitude, as described later.

##### Numerical Simulation

The shear stresses within cast channels with varying diameters at varying flow rates were modeled using COMSOL Multiphysics 5.6 software. The channel walls were selected to be solid boundaries with no slip, and the flow was assumed to be a stabilized laminar flow of water (with a density of 1 g.cm^−2^, and a viscosity of 1 mPa.s). To evaluate the shear stresses at different flow rates, the measured diameter at set flow rates was used as an input for the model.

##### Statistical Analysis

All graphs, curve fitting, and statistical analysis were performed on OriginPro software. Methodology, sample size, means, standard deviations, and significant differences (one‐way ANOVA) corresponding to each analysis are described in the associated figure captions and/or experimental subsections.

## Conflict of Interest

The authors declare no conflict of interest.

## Supporting information

Supplementary Material

## Data Availability

The data that support the findings of this study are available from the corresponding author upon reasonable request.
