# Supplementary material for: Mechanically Tunable Biofabricated Channels Enable Mimicking Arterial Pulsatility and Dynamic Tissue Actuation
Source: Small Sci. 2025 Jun 30;5(9):2500176. doi: 10.1002/smsc.202500176 (PMC12412620; doi:10.1002/smsc.202500176)
Supplement: Supplementary file 1 — Supplementary Material [file SMSC-5-2500176-s001.pdf]

Supporting information

Cécile Bosmans[1,#], Malin Becker[1,#], Liliana S. Moreira Teixeira[2], and Jeroen Leijten[1]*

[1]Leijten Lab, Dept. of BioEngineering Technologies, TechMed Centre, Faculty of Science and Technology, University of Twente, 7522 NB, The Netherlands

[2]Dept. of Advanced Organ bioengineering and Therapeutics, TechMed Centre, Faculty of Science and Technology, University of Twente, 7522 NB, The Netherlands

# These authors contributed equally to this work. Author sequence randomly determined.
* Corresponding author: jeroen.leijten@utwente.nl

Rheology as well as constant perfusion were performed on low, medium and high-DS ATA channels under a low-crosslinking condition, in the same way the experiments were performed in the high-crosslinking condition showcased in the main text. Here, the low-crosslinking condition does not show as clear differences as the high-crosslinking condition in between the differently substituted ATA but does offer another degree of tunability in the material's behavior.

1. Stress-strain curves and rheology Low-CR

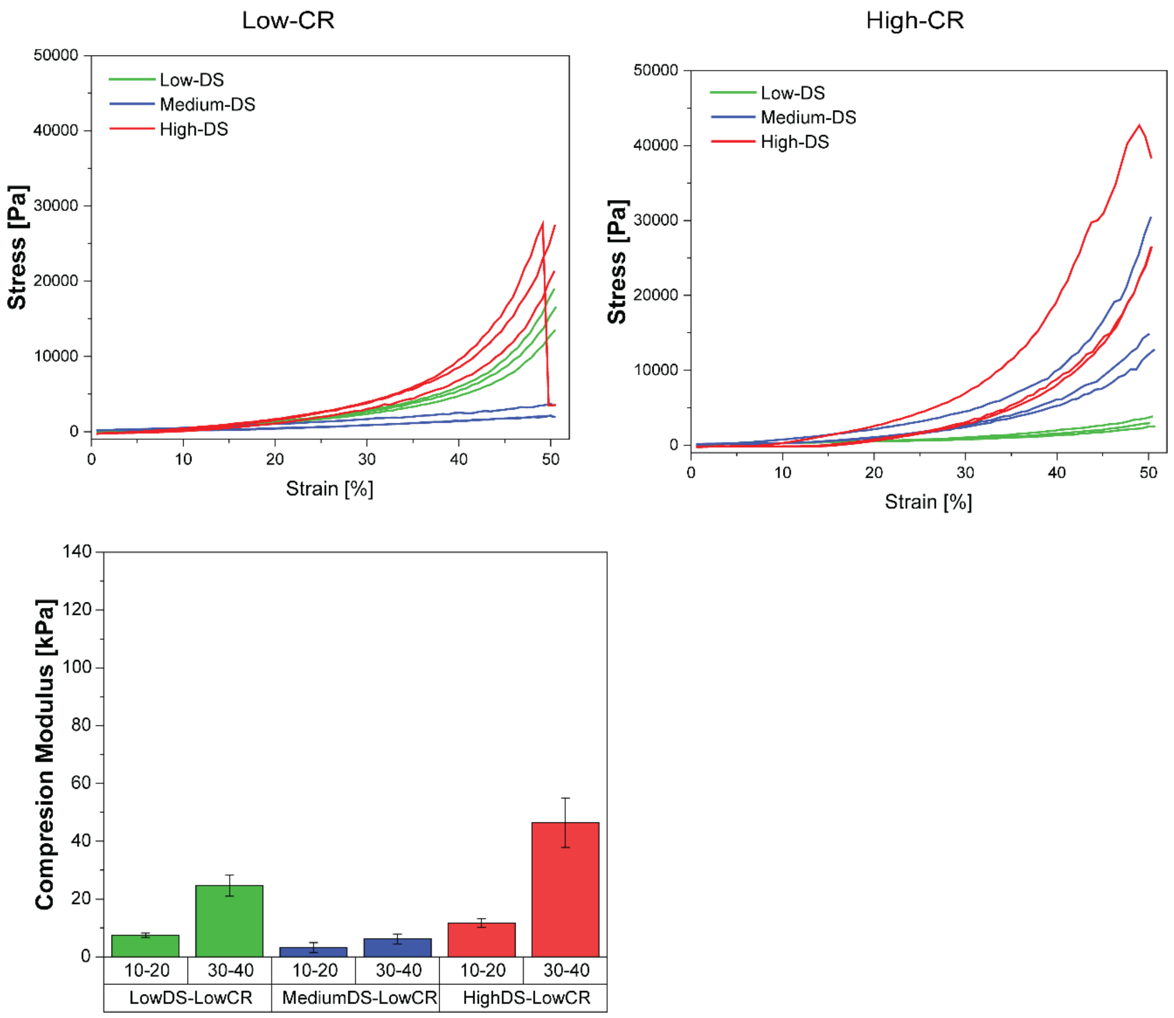

Figure S 1: Stress-strain curves of all conditions and compression of ATA crosslinked at low concentration calculated between 10%-20% compression (Mean ± SD, n=3 for each condition) or 30%-40% compression (Mean ± SD, n=3 for each condition).

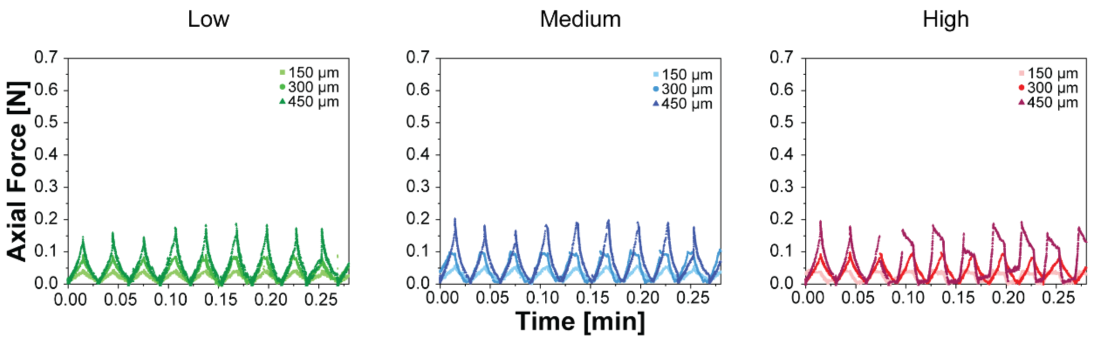

Figure S 2: Cyclic compression of ATA crosslinked at low concentration showing only minor differences between the hydrogels (n=3 channels for each condition).

2. Dilation Low CR

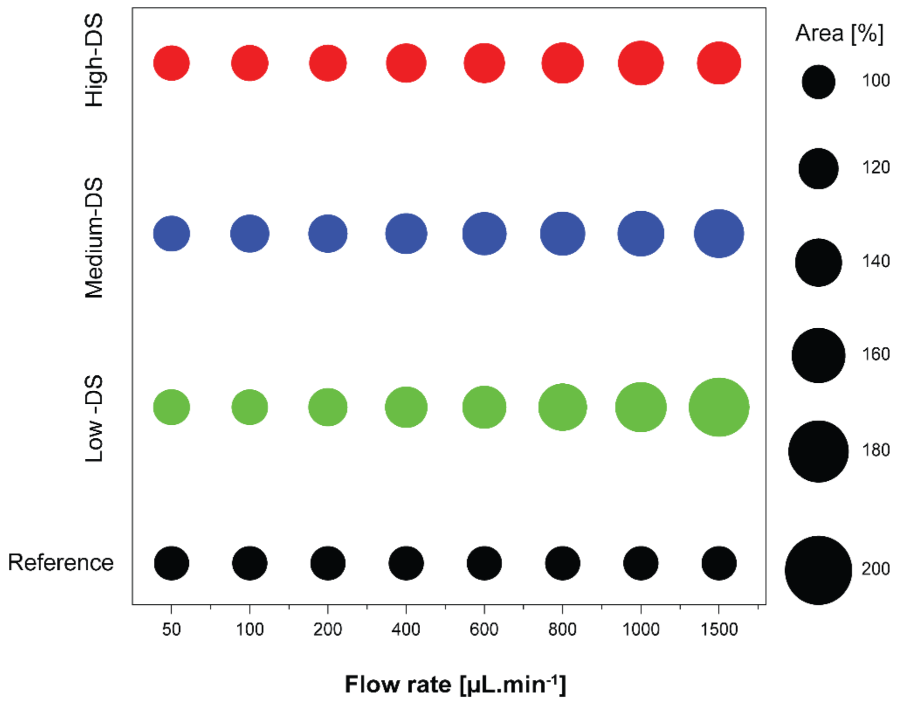

Figure S 3: Channel area increases in response to constant perfusion in the case of the low crosslinking condition (n=3 channels for each condition).

3. Velocity Low CR

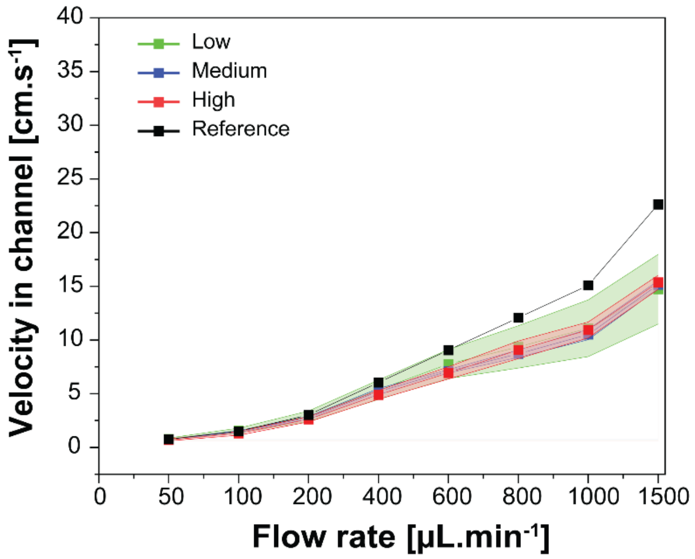

Figure S 4: Velocity at which the channel with low-crosslinking condition is perfused, based on the measured channel cross-section geometry and input flowrate (Mean ± SD, n=3 channels for each condition).

4. Suction

The system was filled with the perfusate and connected to a filled reservoir. It was then recorded and sequentially perfused (pulling from the inlet) at a set steady flowrate for 20 seconds then 15 seconds recovery at 50, 800, 1000, 1500, and 2000 µL/min. The diameter of the channel was consequently measured based on the average of 10 measurement points per flowrate and per technical replicate, averaged per condition. From this, we could also calculate the area modification of the channel when flowed in comparison to the not-flowed channel dimensions. As the applied suction compresses the channel, the diameter shrinks with increasing flow rate for all conditions. This was more strongly observed for the low-DS hydrogels, which is in line with the perfusion results.

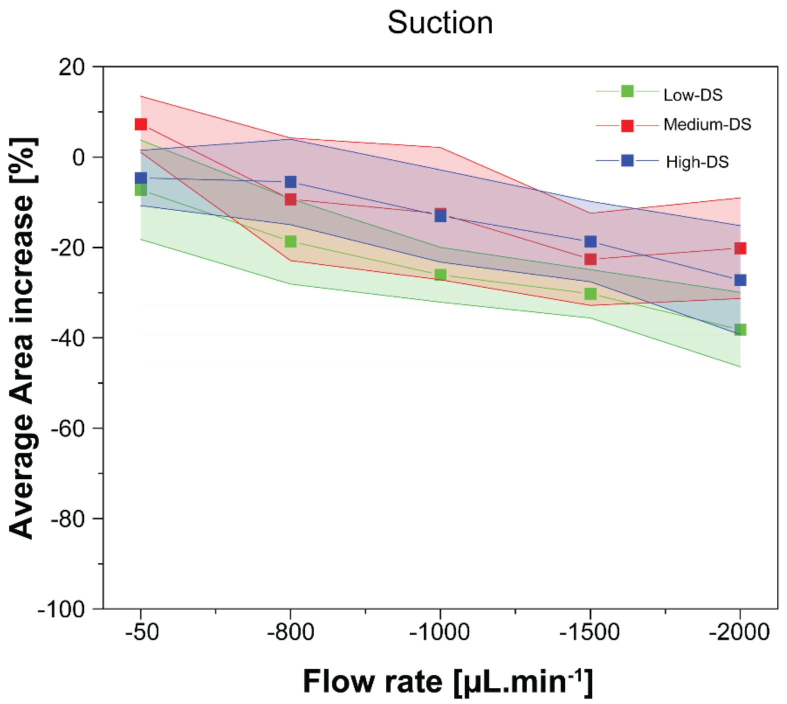

Figure S 5: Average area increase in dependence on the flow speed applied by suction, showcasing that the area decreases with increasing suction, and showing a more pronounced effect for the low DS condition (Mean ± SD, n=3 channels for each condition).

5. Large channel:

The system was recorded and sequentially perfused in the same way as the described steady perfusion. The channel was cast around a larger needle of 21 gauge (819 µm outer diameter). The diameter of the channel was consequently measured based on the average of 10 measurement points per flowrate and per technical replicate, averaged per condition. From this, we could also calculate the area modification of the channel when flowed in comparison to the not-flowed channel dimensions. Compared to the smaller channels discussed in **Error! Reference source not found.**, the larger channels showed less dilation within the same materials and same applied flow rate. This can be potentially correlated to the inverse relationship of radius and pressure, where lower pressures are expected in larger channels, hence, leading to less dilation.

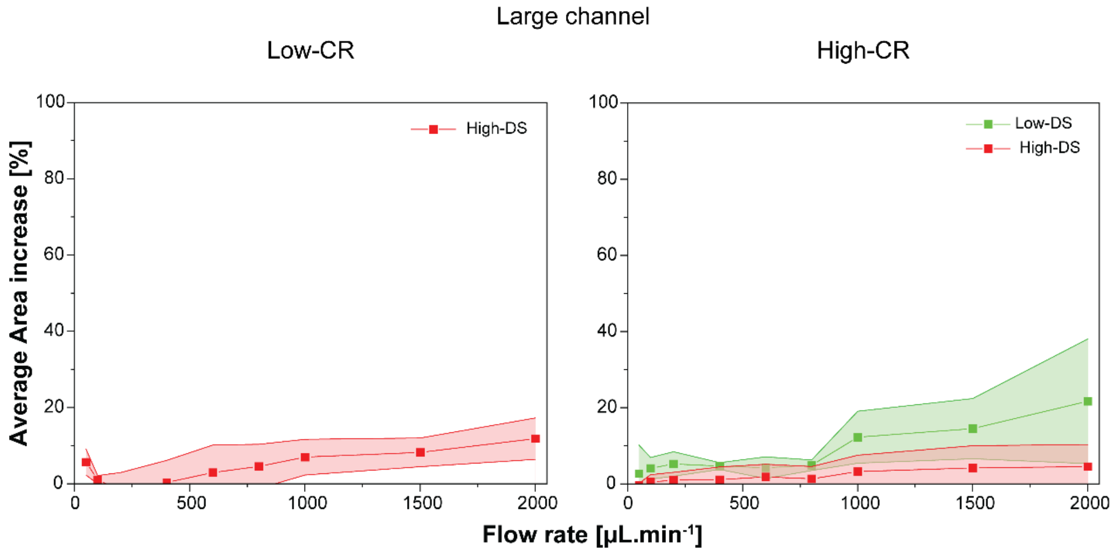

Figure S 6: Average area increase of a large diameter (860 µm) channel cast in low-CR (left, mean ± SD, n=3 channels) and high-CR (right, mean ± SD, n=3 channels for each condition) hydrogels.

6. Velocity profile

The velocity profile obtained for a channel containing a saccular aneurysm reveals not only the circular motion within the cavity, but also showcases, how tissue surrounding the channel can locally be exposed to larger forces due to directed flows against the channel wall (see black arrow).

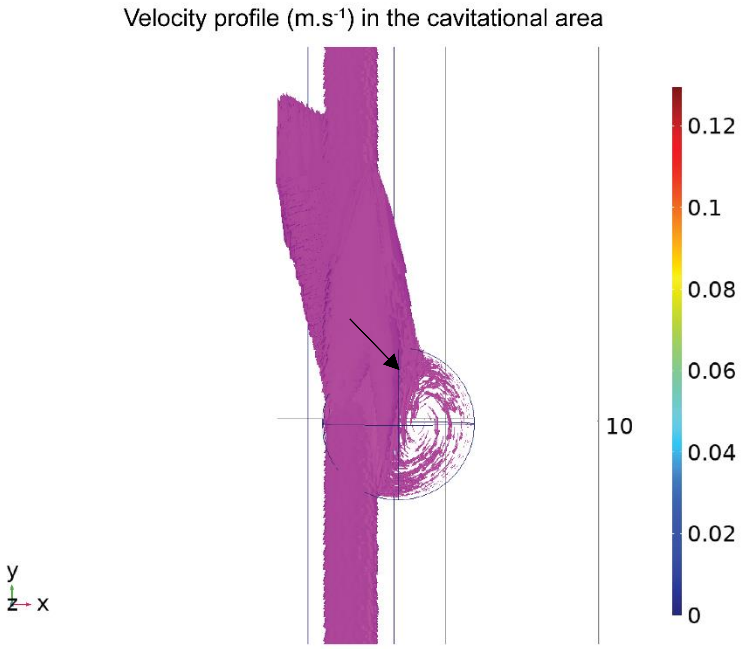

Figure S 7: Velocity profile obtained from COMSOL modeling of a cavity attached to a channel showcasing the circular flow in the cavity and the flow against the opposite channel wall.
